# Self-management perceptions and needs in patients with thoracic surgical wound dehiscence: a descriptive phenomenological qualitative study

**DOI:** 10.3389/fpubh.2026.1948457

**Published:** 2026-09-30

**Authors:** Xuemei Xie, Rong Zheng, Ping Lu, Yujing Zhou, Suxia Shi

**Affiliations:** 1 School of Medicine, Tongji University, Shanghai, China; 2 Shanghai Armed Police Corps Hospital, Shanghai, China; 3 Shanghai Pulmonary Hospital Affiliated to Tongji University, Shanghai, China

**Keywords:** needs assessment, qualitative research, self-management, surgical wound dehiscence, thoracic surgery

## Abstract

**Background:**

Postoperative wound dehiscence is a severe complication in thoracic surgery, yet patients’ self-management experiences remain largely underexplored. This study aims to explore the current cognitive status and potential needs of patients with dehiscence after thoracic surgery regarding self-management in the Chinese context, and to provide a theoretical basis for formulating personalized and precise wound self-management support plans.

**Methods:**

This study adopts a descriptive phenomenological qualitative research design. By using the purposive sampling method, patients with postoperative wound dehiscence in thoracic surgery who visited three tertiary hospitals in Shanghai from March 2026 to May 2026 were selected as the participants for semi-structured in-depth interviews. With the aid of NVivo 15.0 software, the interview data were sorted and analyzed by using the seven-step analysis method of Colaizzi phenomenology.

**Results:**

A total of 21 participants were ultimately included in this study, and 2 core themes and 6 sub-themes were extracted. Theme 1, Cognitive and practical limitations in self-management, comprises: knowledge deficit and cognitive biases; limited self-care capacity; and illness uncertainty with negative attitudes. Theme 2, Multidimensional needs for self-management support, includes: professional knowledge and skills guidance; continuous professional care; and psychosocial support.

**Conclusion:**

Patients with thoracic surgical wound dehiscence experience significant cognitive dissonance and face complex practical barriers during self-management. Their lived experiences highlight an urgent need for tailored professional guidance, continuous care, and psychosocial support. However, recruiting participants exclusively from tertiary hospitals in a single metropolitan area may limit the transferability of these findings. In the future, clinical medical staff should take patients’ needs as the orientation, combine the cultural characteristics of China, and build a full-cycle self-management support model to empower patients, alleviate illness uncertainty, and promote wound healing.

## 1 Introduction

Thoracic surgery is the core approach for treating major chest diseases such as those of the lungs, esophagus and mediastinum (1). Globally, and particularly in China, the volume of thoracic surgeries has surged due to the persistently high incidence of these malignancies. In recent years, despite the rapid development of minimally invasive techniques, poor postoperative wound healing or even wound dehiscence (WD) remains a common and severe complication (2). Thoracic incisions are usually accompanied by high tension from continuous respiratory movements. When combined with underlying risk factors such as malnutrition, diabetes, or long-term smoking, the incidence of WD significantly increases (3). Relevant studies have shown (4–6) that the incidence of dehiscence in abdominal surgeries in general surgery is 0.5–3%, and that in thoracic surgeries in cardiac surgery is 0.3–10%. This not only delays the recovery process of patients, reduces their quality of life, increases the risk of infection and the rate of rehospitalization, but also brings great physical pain and deep psychological trauma to patients. During the prolonged rehabilitation of WD, in addition to professional clinical interventions, effective self-management by the patient plays a crucial role. Self-management of thoracic WD is a multidimensional process that closely relies on the active participation of patients and their families, covering daily home wound care, pain monitoring, respiratory function exercises, and the early identification of infection signs (7). However, implementing effective self-management in the Chinese cultural context faces unique barriers. Influenced by traditional concepts, Chinese patients exhibit a high degree of psychological dependence on healthcare professionals. When facing open wounds, they often show extreme panic, stigmatization due to wound exudation or unpleasant odors, and a misconception that self-intervention might impede healing (8). Furthermore, the Chinese healthcare recovery model is highly reliant on family caregivers. The lack of professional wound care skills among family members, coupled with the fragmentation of rehabilitation guidance after discharge, often exacerbates both the patient’s anxiety and the caregiver’s helplessness, leading to poor compliance and inefficient home management (9). Although the active participation of patients is crucial for wound healing, few studies have delved into their subjective cognition and genuine self-management needs after the wound splits from the perspective of patients. Therefore, this study adopts a qualitative research method and conducts semi-structured in-depth interviews, aiming to explore the current cognitive status and potential needs of patients with dehiscence after thoracic surgery regarding self-management in the context of Chinese medical culture. To provide a theoretical basis for formulating personalized and precise wound self-management plans for patients, promote wound healing and improve their quality of life.

## 2 Methods

### 2.1 Study design and participants

This study adopted a descriptive phenomenological qualitative research design. Through semi-structured in-depth interviews, it explored the current cognitive status and needs of patients with dehiscence after thoracic surgery regarding self-management during the rehabilitation period. To ensure the depth and breadth of the research results, this study adopted purposive sampling and selected patients with WD who were hospitalized in the thoracic surgery department or visited the wound and stoma outpatient department of three tertiary hospitals in Shanghai from March 2026 to May 2026 as the participants. The entire research process strictly adhered to the Qualitative Reporting Standards (COREQ) guidelines (10) to ensure the rigor, transparency and reproducibility of the research process.

#### 2.1.1 Inclusion and exclusion criteria

Inclusion criteria: (1) Adult patients aged 18 years or older; (2) Those who have undergone thoracic surgery in the past (such as median thoracotomy, lateral thoracotomy, or thoracoscopic-assisted surgery) and are currently experiencing unhealed poor incision healing or WD (including superficial dehiscence or deep tissue dehiscence) at the time of the interview; (3) The course of wound intervention is≥1 week, and the patients are undergoing active wound care or treatment; (4) Clear consciousness, with normal language expression, communication and comprehension abilities; (5) Understand the purpose and significance of this study, voluntarily participate and sign the informed consent form.

Exclusion criteria: (1) Those in a critical stage of illness, such as those with severe sepsis, requiring mechanical ventilation, etc., and unable to cooperate with the interview; (2) Those with severe mental system disorders or cognitive dysfunction; (3) Those with other serious physical complications such as heart, liver and kidney problems that make it impossible for them to tolerate long-term interviews. (4) Those who voluntarily request to withdraw during the interview or provide extremely incomplete information.

#### 2.1.2 Sampling process and data saturation

The sample size of this study was determined based on the principle of information saturation, that is, saturation was reached when no new topics or viewpoints emerged in three consecutive interviews (11). Purposive sampling with a maximum variation strategy was employed, actively ensuring diversity in patients’ age, gender, surgery type, wound severity, and educational level. Regarding the sampling process, a total of 28 patients who met the initial clinical screening criteria were approached. Among them, 4 patients refused to participate due to severe wound pain or privacy concerns, and were thus transparently excluded for not meeting the qualitative willingness criterion. Additionally, pre-interviews were conducted with 3 patients to pilot-test the scientificity, rationality, and feasibility of the interview guide. Because these were pilot tests rather than formal data collection, their data were excluded from the final research results. This left a cohort of 21 patients for the formal in-depth interviews. To rigorously assess data saturation, data collection and analysis were conducted concurrently. After each interview, the researchers immediately transcribed the recordings and conducted preliminary coding. Starting from the 19th participant, no new themes or viewpoints regarding the cognition and needs of wound self-management emerged in three consecutive interviews. Following a comprehensive discussion, the research team unanimously agreed that data saturation had been achieved, and recruitment was subsequently halted. Ultimately, 21 participants were included in the final analysis.

### 2.2 Data collection method

Potential participants who met the inclusion criteria were purposively approached in the thoracic surgery department and the wound and stoma outpatient clinic. Researchers introduced the purpose, significance and privacy confidentiality principles of the study to eligible patients in plain and neutral language. Given that a split wound not only causes physical pain but also often comes with a high sense of stigma, researchers spend about 15–20 min in empathetic communication with the patient before the formal interview to build a solid trust relationship and lower the patient’s psychological defense. The interview process is conducted in a quiet ward or an independent consultation room in the outpatient department, adopting a one-to-one and a half structured format. Before the interview, general sociodemographic and clinical data of the patients were collected, such as age, underlying diseases, type of surgery, and duration of wound dehiscence, etc. The interview was jointly conducted by two researchers who had received qualitative research training. One of the researchers adopted open-ended and exploratory questioning, while the other was responsible for observing and recording the patients’ non-verbal information, such as emotions, changes in facial expressions, body movements and other non-verbal information. The entire process was recorded with the patient’s consent. Timely on-site verification should be conducted for ambiguous, contradictory or unclearly expressed content to ensure the authenticity, accuracy and completeness of the information. The entire interview process lasts for 25–49 min. Within 24 h after the interview, two researchers transcribed the audio verbatim into text. After the transcription is completed, the text is returned to the respondents through member verification to confirm whether the expression is accurate and there are no omissions. Patients can modify or delete the content. Finally, the research team conducts the final proofreading.

### 2.3 Interview guide

Based on the purpose and theme of this study, the research team has initially developed a semi-structured interview guideline. To ensure the scientific nature, pertinence and clinical relevance of the interview guidelines, five experts were invited for consultation by letter, including two thoracic surgeons, two wound specialist nurses and one qualitative research expert. Five experts put forward independent suggestions regarding the comprehensiveness of the interview guidelines, the accuracy of the expression and whether there was any inducement. Based on the feedback from experts, the team discussed and optimized the outline through an online meeting. Subsequently, pre-interviews were conducted with three patients. Based on their feedback, several specific adjustments were made to refine the interview guide. First, adjust the logical sequence of the questions, starting from the patient’s physical pain experience and its impact on daily life, and then delve into specific self-care behaviors. This is helpful in reducing the patient’s psychological defense. Secondly, the wording was modified to use language that is easy for patients to understand instead of medical terms. The core issues are shown in Table 1.

**Table 1:** Interview guide.

Theme	Question
Current status and experiences of the wound	1. How is the current condition of your wound? What impacts does a split wound have on your daily life, mood and sleep?
	2. How did you feel when you found that your postoperative wound had split open??
Perceptions	3. During the wound recovery process, how do you care for this wound (such as changing dressings, observing, and relieving pain, etc.)?
	4. During the long-term recovery process of the wound, what do you think is the most challenging or helpless difficulty for you? How did you deal with it?
	5. Do you think your current knowledge and skills in wound care are sufficient? What are the shortcomings?
Needs	6. During the period of wound healing, what kinds of support and assistance do you hope to receive from medical staff, your family or society?
	7. If future hospitals provide home self-management guidance for wound dehiscence, what specific contents or functions do you hope it will include?
Others	8. Do you have any other ideas or suggestions that you would like to add?

### 2.4 Research team

The research team consists of five experienced nursing experts and postgraduate students. All team members have received systematic qualitative research course training and are proficient in COREQ guidelines and in-depth interview skills. The division of labor within the team was clear, and the interviews and records were carried out by two researchers, respectively. To avoid the influence of reactivity bias, the researchers who led the interviews were all direct nurses in charge of the patients who were not interviewed. In addition, the research team holds weekly seminars to jointly review the problems encountered during the coding process. When there are differences in coding or topic summary and no consensus can be reached, the final ruling shall be made by the corresponding author with rich clinical experience in thoracic surgery and a background in wound management to ensure the scientific and accurate nature of the research results.

### 2.5 Data analysis method

The NVivo 15.0 qualitative data analysis software is adopted to assist in the management and analysis of text. Data processing strictly adopted the seven-step phenomenological analysis method of Colaizzi (12). The specific steps include: Researchers repeatedly and carefully read all the transcribed texts to obtain an intuitive perception of the patient’s overall experience; Extract meaningful statements related to the cognition and needs of wound self-management; Initially code these statements; Cluster the codes with similar semantics to form sub-topics and main topics; Then provide a detailed description of the extracted topic; On this basis, the cognitive and demand framework for self-management of patients with dehiscence in thoracic surgery is extracted. Finally, the summarized themes and descriptions were sent back to the 10 respondents for verification. They were asked whether these themes and contents were in line with their true cognitive status and needs, and whether there were any omissions or new themes. The entire coding process was independently completed by two researchers. For inconsistent encoded content, the research team will discuss together until a 100% consensus is reached. Finally, the extracted themes, sub-themes and quotations were independently translated into English by two bilingual researchers to ensure semantic equivalence.

### 2.6 Rigor

This study strictly adhered to the COREQ guidelines for qualitative research (10). Quality control is carried out from four dimensions: Credibility, Dependability, Confirmability and Transferability to minimize subjective bias to the greatest extent.

Credibility: Triangulation was achieved by having two researchers independently code and analyze the data. Furthermore, detailed member-checking was conducted. The verbatim transcripts and the extracted themes were returned to 10 participants via face-to-face follow-up visits or encrypted WeChat communications. Participants were asked to confirm whether the synthesized findings accurately reflected their true lived experiences. All 10 participants confirmed the accuracy of the themes without requesting any structural modifications or deletions, thereby solidifying the credibility of the data.

Dependability: Properly preserve all research interview materials, including original audio, verbatiword transcripts, codebooks, expert consultation records, and team meeting minutes, to form complete review materials and ensure the repeatability of research methods.

Confirmability: To reduce researchers’ subjective tendencies, researchers write reflection logs throughout the data collection and analysis process, recording possible biases during interviews, and proactively disclose and jointly analyze them in group discussions, promptly formulating improvement measures. Meanwhile, the results section extensively quotes the original statements of the respondents to confirm that the conclusion is directly derived from the patient data.

Transferability: In the results section, the demographic characteristics of the interviewed patients, the clinical background of the wounds, and the specific research environment are detailedly reported to enable readers to assess the applicability of the results of this study in other geographical regions or medical contexts.

### 2.7 Ethical considerations

This study has been approved by the Medical Ethics Committee of Shanghai Pulmonary Hospital (Ethics Approval Number: Q25-840Y). The entire research process strictly adheres to the ethical principles of medical research involving human subjects in the Declaration of Helsinki, upholding the principles of autonomy, voluntariness, confidentiality and harmlessness. Before the formal data collection, the researchers fully explained to all the interviewed patients the purpose, process, interview content and privacy protection of this study. All respondents signed the informed consent form with full knowledge. Furthermore, the researchers explicitly promised the participants that they had the right to unconditionally withdraw from the interview at any stage of the study. The decision to withdraw would in no way affect their subsequent clinical diagnosis, wound dressing change treatment, or normal doctor-patient relationship. Once the patient withdraws, all their data will be immediately and completely destroyed. To protect the privacy and legitimate rights and interests of patients to the greatest extent, all interview recordings, transcribed texts and clinical data of patients have undergone strict desensitization and anonymization processing.

## 3 Results

A total of 21 patients with dehiscence after thoracic surgery who met the criteria were included in this study, including 13 males and 8 females. The age ranged from 45 to 78 years old, with an average age of 62.48 years old. In terms of primary diseases, lung cancer and esophageal cancer are the most common, accounting for 47.6 and 23.8%, respectively. The dehiscence sites covered traditional lateral thoracotomy incisions, median sternal incisions, and small incisions assisted by thoracoscopy. The time for WD ranged from 10 to 52 days, with an average course of WD of approximately 21.33 days. Surgical approaches included lateral thoracotomy (*n* = 10), median sternotomy (*n* = 4), and video-assisted thoracoscopic surgery (VATS) (*n* = 7). Notably, while open incisions caused greater physical pain during coughing, patients across all surgical types experienced equally profound psychological panic and uncertainty upon discovering the dehiscence. The specific sociodemographic characteristics of the participants are detailed in Table 2.

**Table 2:** General information of participants.

Category	Grouping	Frequency (n)	Percentage (%)
Gender	Male	13	61.90
	Female	8	38.10
Age (year)	<60	8	38.10
	60 ~ 70	8	38.10
	>70	5	23.81
Educational level	Primary school	6	28.57
	Middle school	11	52.38
	Undergraduate	4	19.05
Residence	Urban	9	42.86
	Town	6	28.57
	Rural	6	28.57
Employment status	Employed	6	28.57
	Farmer	5	23.81
	Self-employed	2	9.52
	Retired	8	38.10
Primary disease	Lung cancer	10	47.62
	Esophageal cancer	5	23.81
	Mediastinal tumor	4	19.05
	Pulmonary bullae	1	4.76
	Severe chest trauma	1	4.76
Splitting time (day)	<10	4	19.05
	10 ~ 20	8	38.10
	>20	9	42.86
Patient source	Inpatient	5	23.81
	Outpatient	16	76.19
Occurrence of infection	Yes	8	61.90
	No	13	38.10

### 3.1 Theme 1: cognitive and practical limitations in self-management

#### 3.1.1 Bodily vulnerability and fear-driven cognitive misconceptions

Patients with thoracic surgical WD experience profound fear and cognitive dissonance during their home rehabilitation. Rather than a simple lack of information, their lived experiences are dominated by intense bodily vulnerability and terror of further physical damage. For instance, the severe tearing tension of thoracic wounds often translates into a deep-seated fear, causing patients to misinterpret normal physiological processes or rehabilitation requirements. Driven by this existential fear, patients often develop cognitive biases and adopt fear-driven maladaptive behaviors, such as resisting necessary coughing or early ambulation out of sheer terror of wound deterioration.

*P13: The nurse said I should cough more to expel phlegm, but whenever I cough, I feel as if my chest is being torn apart. I always feel that the wound split open because I coughed too hard a few days ago. Now I’d rather swallow the phlegm than move it.*

*P3: I had no idea how this wound could be considered healed. There was a bit of yellowish water on the gauze. I thought it was pus and had roamed inside. I was so scared that I asked my son to take me to the emergency room in the middle of the night. But the doctor said it was normal exudate.*

*P15: I thought the more I moved, the more the flesh would tear open. I stayed literally frozen in bed for three days, not daring to breathe deeply. I did not know that staying still was actually making my lungs worse.*

#### 3.1.2 Physical barriers and a sense of powerlessness in self-care

When managing themselves at home, patients are confronted with a profound sense of powerlessness, exacerbated by physical and anatomical limitations. Because thoracic surgical wounds are mostly located in blind spots (e.g., the lateral or posterior chest wall), patients face severe physical struggles in observing and changing dressings independently. This physical barrier strips them of their sense of control. Furthermore, the lack of continuous professional guidance translates into deep frustration and anxiety during daily care routines, leading to a constant dread of causing secondary infections.

*P7: The wound is on the side of my back. I can neither see nor reach it myself. My wife is getting old and her hands are shaking. Every time she changes my dressing, it makes me hurt a lot. The bandage is also all wrinkled. I’m always afraid that bacteria might get in and cause inflammation.*

*P6: Before being discharged from the hospital, the nurse demonstrated the dressing change steps once. When I got home and tried to do it myself, I was in a flustered state, always feeling that I wasn’t doing it right and fearing that it might cause an infection.*

#### 3.1.3 Existential uncertainty and emotional exhaustion

Patients with cracked wounds often fall into a sense of uncertainty about their illness, which in turn leads to negative attitudes such as anxiety, shame and pessimism. Thoracic surgical wounds heal slowly and are often accompanied by recurrent exudation and pain. Due to the inability to predict when the wound will heal and whether the current intervention will be effective, this long-term sense of uncertainty seriously consumes the psychological energy of patients, causing them to lose confidence in recovery. Especially for patients with concurrent malignant tumors, wound problems further intensify their pessimism about the prognosis of cancer. In addition, the unpleasant smell from wounds and the sense of shame brought about by long-term bed rest also make them resist and actively participate in self-management.

*P18: I thought I was about to make it after the surgery, but it split open again. I felt extremely unlucky and had no confidence that it would get better soon, so I just kept waiting day by day.*

*P10: Every time I go to change my dressing, I’m on edge, not knowing if it’s getting better or worse today. The smell from the wound was very unpleasant. I felt that I could do nothing about it now and had no energy to deal with it all day long.*

### 3.2 Theme 2: multidimensional needs for self-management support

#### 3.2.1 Needs for professional knowledge and skill guidance

Patients with cracked wounds urgently need medical staff to provide systematic knowledge on wound healing and practical guidance on home care skills that can be operated. Patients generally reported that the oral education at discharge could not meet the actual needs of self-management at home. At the knowledge level, they hope to obtain popular science about the specific criteria for determining the stages of wound healing and abnormal characteristics, such as the volume and color of exudate. In terms of skills, patients urgently need practical guidance, including the standard steps for aseptic dressing changes at home, the correct techniques to protect the tension of the incision when coughing, and the specific movements for getting up and lying down safely. In addition, patients with underlying diseases such as diabetes and chronic obstructive pulmonary disease also hope to receive professional guidance on the coordinated management of comorbidities and clarify the connection between the control of underlying diseases and wound healing.

*P5: The nurse said to keep the wound clean and dry, but to what extent is it considered dry? How much fluid has seeped that requires going to the hospital? I hope there could be a detailed booklet telling me when not to panic and when I must see a doctor.*

*P16: I have diabetes. I do not know at what level my blood sugar should be controlled for the best wound healing. Also, no one has given me a definite answer about what I can and cannot eat in my diet.*

#### 3.2.2 Needs for continuous professional care support

The interview found that for some patients, professional care support came to an abrupt end after discharge, which made them feel helpless when facing sudden wound conditions. Some patients living in rural or urban areas find it rather troublesome to go to tertiary hospitals for regular follow-up visits due to inconvenient transportation. Some older adults, those with limited mobility and those with severe wounds hope to obtain convenient channels for home dressing changes or referrals from community health service centers to reduce the burden of traveling to and from the hospital. Meanwhile, some patients also expressed their hope to obtain standardized wound assessment tools for self-monitoring and record-keeping at home, which could serve as a reference for medical staff during follow-up visits.

*P4: In the hospital, the nurses were always there. But at home, I suddenly felt completely at a loss. When the wound got a bit red, I did not even know who to call… It would be a lifesaver to have a direct online channel to ask experts at any time.*

*P20: The people at our village clinic dare not take on the wounds after such surgeries, saying they are afraid of infection. Every time I change my dressing, I have to take over an hour by car to a larger hospital. I hope there can be a door-to-door dressing change service, or the nurses in the town can coordinate and guide the doctors from the big hospital.*

#### 3.2.3 Needs for social support and psychological adjustment

During the long-term care process of a wound opening, the patient’s need for social support and psychological adjustment gradually becomes prominent, covering multiple aspects such as family support, peer support, and professional psychological support. In terms of social and family support, as Chinese patients are highly dependent on family care, they urgently hope that medical staff can provide psychological comfort and skills guidance to the main caregivers to alleviate the panic of family members. In terms of peer support, patients are eager to communicate with fellow patients who have had similar experiences and successfully recovered, to gain experience and rebuild confidence. In terms of psychological adjustment, some patients with severe negative emotions and a long course of illness explicitly stated that they need psychological counseling and support to help them adjust their mindset and get out of a pessimistic and negative state.

*P8: No one around me has ever had this disease, and I have no idea how others got through it. If there were a patient support group to exchange experiences, I would feel more at ease.*

*P21: Sometimes, just thinking about it makes me feel extremely depressed. It just will not get better. If only someone could offer some guidance and help me adjust my mindset.*

*P7: It’s always my wife who changes my dressing for me. She does not understand either. Every time she’s very careful, afraid of hurting me. It would be great if she could also teach the family how to change it and how to observe.*

## 4 Discussion

### 4.1 Insufficient cognition of self-management among patients with thoracic surgical WD

This study found that patients with thoracic surgical WD generally have serious cognitive biases in self-management and are accompanied by a strong sense of disease uncertainty. From the perspective of a conceptual pathway, these cognitive limitations act as the primary trigger that subsequently translates into practice barriers. This phenomenon can be deeply explained by Mishel’s uncertainty in illness theory (13). This theory points out that when patients lack clear information about their diseases, it is difficult for them to make accurate predictions about symptoms and prognosis, thereby leading to cognitive dissonance and negative coping. Unlike conventional chronic wounds such as diabetic foot, dehiscence of external chest wounds is a sudden postoperative complication. More importantly, thoracic wounds bring unique physiological challenges, specifically the continuous chest expansion during breathing and the extreme tearing tension caused by coughing. When confronted with the reality of a long and unpredictable healing period, this intense sense of “uncertainty” directly leads to patients’ panic over normal exudate and their adoption of incorrect protective behaviors such as “absolute bed rest and refusal to expectorate” out of fear of WD. Teklemariam et al. (14) also confirmed that patients with postoperative abdominal wounds have equal difficulty in identifying infection precursors. However, the cognitive dilemmas exhibited by thoracic surgery patients in this study were more severe. This is mainly because in the context of traditional Chinese medical culture, patients hold traditional views on medical authority, “focusing on treatment while neglecting care”, highly rely on the authoritative intervention of medical staff (15), and experience severe stigmatization and shame due to wound exudate and odor. Furthermore, research has found that older adults often deliberately conceal the deterioration of wound symptoms from their adult children to avoid becoming a burden to them. This lack of transparent family communication seriously dampens their willingness to actively acquire health knowledge, intensifies their inner uncertainty, and often delays timely medical intervention. Clinically, it indicates that the traditional fragmented and oral discharge education can no longer meet the needs of this population. Future clinical interventions should break through the single skill education and construct a “cognitive-psychological” dual-line intervention model based on the theory of uncertainty. While correcting patients’ misunderstandings about rehabilitation, it is necessary to inform them in advance of the characteristics of each stage of wound healing to reduce their fear and shame about the unknown course of the disease, thereby reshaping their intrinsic motivation for self-management.

### 4.2 Multiple challenges in self-management practices among patients with thoracic surgical WD

Patients’ cognitive limitations and fears translate directly into practical self-management barriers, which are further exacerbated by physical limitations and culturally specific social support structures. The inefficiency problems that patients face in wound self-management practices are mainly the result of the interweaving of physiological and anatomical limitations, skill thresholds, and the weak local social support system. From an anatomical perspective, thoracic surgical incisions have the special characteristics of high tension and concealed location, which leads to significant physiological obstacles for patients to perform aseptic operations under limited visual fields. This is highly consistent with the conclusion proposed by Hackert et al. (16) in their research on limited visibility in chronic wound care is the primary obstacle to self-management at home. Furthermore, this study further reveals the predicament of home care under China’s specific medical system. This study found that there are certain differences between outpatients and inpatients. Outpatients mainly reported facing severe practical challenges, such as difficulty in independently performing aseptic dressing changes and monitoring wound exudation without professional supervision. Under the cultural expectations of traditional filial piety, there exists a serious reliance on family caregivers in the family environment. However, this absolute family responsibility has caused serious emotional and physical damage to both sides. Due to the lack of medical training, nursing staff are under great pressure and burnout, while patients feel deeply guilty. Inpatients, on the other hand, exhibit limited self-management abilities, mainly reflecting high expectations for inpatient care and a deep fear of being discharged soon. In European and American countries, home wound care for patients is usually carried out regularly by mature Community wound care nurses, and patients only need to cooperate with basic observations (17). In contrast, the current home rehabilitation service network in China is still not perfect. Furthermore, cultural differences may cause patients to experience a sense of shame related to the foul smell of wounds, making Chinese patients reluctant to seek help from primary medical institutions. They often regard unhealed wounds as a source of shame and are reluctant to expose them to community nurses or neighbors. Due to the lack of systematic operation training before discharge, and driven by the traditional family dynamic where caregivers act out of excessive caution for fear of exacerbating the patient’s breathing or coughing pain, family members often fail to meet the standard of sterility for fear of causing pain or secondary injury to the patient during the operation. This indicates that there is a huge gap between the current clinical discharge preparation services and the actual needs of patients at home. Under this clinical revelation, merely emphasizing the patient’s own compliance is far from sufficient. In the future, efforts should be made in a coordinated manner from dimensions such as operation assistance tools, standardized training for caregivers, and community services to gradually improve the home-based rehabilitation support system and enhance the standardization of patients’ self-management and the safety of home-based rehabilitation (18, 19).

### 4.3 Addressing the needs of patients with WD to optimize self-management strategies

The cognitive limitations and practical obstacles of the patients have led to their subsequent support needs, which require intervention tailored to their unique physical and cultural challenges. The needs of patients with dehiscence in thoracic surgery for self-management support mainly include professional knowledge and skills guidance, continuous professional care support, psychological, social and family support, etc. Huang et al. (20) conducted a systematic review of patients with surgical wound dehiscence, pointing out that a complete home support system can effectively enhance patients’ self-management compliance and shorten the wound healing cycle. However, in current clinical practice, there are widespread problems such as fragmented support content and poor service connection, which are difficult to meet the needs of patients for home rehabilitation. In this study, patients clearly expressed their demands for professional knowledge and operational skills guidance. Stratified knowledge popularization content and operational norms were set up for patients with different wound types such as surface dehiscence and deep tissue dehiscence, as well as those with different concurrent symptoms such as diabetes and malnutrition. Clarify the criteria for differentiating normal exudate from infection signs, the operation process for aseptic dressing changes at home, and the incision protection techniques during coughing and activities. Specifically addressing the physiological challenges of thoracic wounds, medical staff must clarify the incision protection techniques during coughing and respiratory activities to alleviate patients’ fear of tension-induced tearing. Provide standardized graphic and textual manuals and step-by-step operation videos to make up for the shortcomings of traditional oral education such as fragmentation and homogeneity, and provide a scientific basis for patients’ self-management at home. Meanwhile, patients with dehiscence in thoracic surgery have an urgent need for continuous professional care support. The self-management support system should establish a service system of hospital - community - family, build an online wound consultation and dynamic follow-up platform, and provide wound assessment and question answering for patients (21); For older adults, those with mobility issues, and those from remote areas, community health service centers are linked to undertake regular dressing changes and technical guidance. Standardized home wound monitoring and recording tools are provided to ensure the connection between home rehabilitation data and clinical diagnosis and treatment, thereby enhancing the continuity and standardization of self-management (22). In addition, addressing the unique cultural context of family dependence and stigmatization, patients have also put forward significant demands for social support and psychological adjustment. In the process of optimizing self-management strategies, it is always necessary to put patients at the center, incorporate the main family caregivers into the education and training system simultaneously, enhance the operational skills and psychological support capabilities of caregivers, and reduce the anxiety and burden of family care. In terms of psychological support, a phased emotional guidance module can be set up based on clinical psychological intervention norms. Combined with peer experience sharing and professional psychological guidance, it can alleviate patients’ sense of uncertainty and shame about the disease, correct pessimistic and negative rehabilitation attitudes, and enhance patients’ internal motivation and confidence in participating in self-management.

## 5 Limitations

This study deeply explored the cognitive status and multi-dimensional needs of patients with thoracic surgical wound dehiscence in self-management through qualitative research methods, but there are still certain limitations. Firstly, the sample sizes of this study were all from three tertiary hospitals in Shanghai, with relatively limited sampling areas. Meanwhile, the overall educational level of the patients was relatively high. Compared with other regions or primary medical institutions, these patients usually have relatively abundant medical resources and higher health literacy. Therefore, the results of this study may affect its promotion in other environments. Secondly, as wound dehiscence is often accompanied by a high sense of stigma and severe physical pain, some patients who met the inclusion criteria refused the interview invitation. The self-management predicament of these non-included patients may be even more severe, thus there is a potential selection bias. Thirdly, since the concept of self-management is basically carried out in a family environment, the reactions of hospitalized patients may bring potential biases. Fourth, this study solely focused on the individual perspective of the patients. Given the highly family-dependent nature of the Chinese healthcare recovery model highlighted in our findings, the omission of primary family caregivers’ perspectives is a significant limitation, as the interdependent dynamic between the patient’s anxiety and the caregiver’s burden remains underexplored. Future research is highly recommended to employ a dyadic qualitative approach, simultaneously exploring the shared lived experiences and mutual support needs of both patients and their family caregivers. Finally, the interview only captured the patient’s cognition and needs at a specific time point when the wound split open. However, wound healing is a long and dynamic physiological and psychological process. The patient’s sense of uncertainty about the disease, needs and coping strategies may change as the recovery process progresses. In the future, multi-center and cross-regional mixed qualitative and quantitative research can be carried out to enhance the extrapolation and universality of research results.

## 6 Conclusion

This study, through qualitative research methods, explored the current cognitive status, practical predicaments and support needs of patients with dehiscence after thoracic surgery regarding self-management. Studies have found that patients with thoracic surgical WD have a relatively low overall cognitive level of self-management. They generally have problems such as fragmented disease knowledge and cognitive biases in rehabilitation, and are accompanied by a strong sense of disease uncertainty and a negative rehabilitation mentality. Meanwhile, patients’ self-management practices are confronted with multiple practical obstacles, which restrict the effective implementation of patients’ self-management. Therefore, patients have diverse demands for self-management, including professional knowledge and practical skills guidance, continuous care support, social psychological and family care, etc. In the future, a full-cycle self-management support model should be constructed based on the needs of patients, effectively enhancing their self-management capabilities, promoting wound healing, and improving their rehabilitation outcomes and quality of life. At a policy and institutional level, it is highly recommended to integrate digital support platforms and structured home-based follow-ups into standard discharge protocols. Such policy integrations will effectively bridge the critical gap between hospital care and home rehabilitation, ensuring the continuity and safety of patients’ self-management.

## Data Availability

The original contributions presented in the study are included in the article/Supplementary material, further inquiries can be directed to the corresponding author.

## References

[1] Kim AW Jaklitsch MT. The evolving landscape of thoracic surgical oncology. J Surg Oncol. (2023) 127:217–20. doi: 10.1002/jso.27174, 36630095 PMC10107667

[2] Morrell Scott N Lotto RR Spencer E Grant MJ Penson P Jones ID. Risk factors for post sternotomy wound complications across the patient journey: a systematised review of the literature. Heart Lung. (2022) 55:89–101. doi: 10.1016/j.hrtlng.2022.04.013, 35504241

[3] Cai YS Li XY Ye X Li X Fu YL Hu B . Preoperative controlling nutritional status score (CONUT) predicts postoperative complications of patients with bronchiectasis after lung resections. Front Nutr. (2023) 10:1000046. doi: 10.3389/fnut.2023.1000046, 36742422 PMC9895366

[4] Lozada Hernández EE Hernández Bonilla JP Hinojosa Ugarte D Magdaleno García M Mayagoitía González JC Zúñiga Vázquez LA . Abdominal wound dehiscence and incisional hernia prevention in midline laparotomy: a systematic review and network meta-analysis. Langenbeck's Arch Surg. (2023) 408:268. doi: 10.1007/s00423-023-02954-w, 37418033

[5] Reifegerste CP Moreira BL Arakaki RA Medeiros AK. Expected imaging findings and postoperative complications of median sternotomy. Radiol Bras. (2025) 58:e20240094. doi: 10.1590/0100-3984.2024.0094, 39975587 PMC11839153

[6] Chen Y You Y Zhang Q Cao Q. A commentary on concerns regarding 'metabolic syndrome and surgical complications: a systematic review and meta-analysis of 13 million individuals'. Int J Surg. (2024) 110:8169–70. doi: 10.1097/js9.0000000000001497, 39806753 PMC11634072

[7] Wang YQ Liu X Jia Y Xie J. Impact of breathing exercises in subjects with lung cancer undergoing surgical resection: a systematic review and meta-analysis. J Clin Nurs. (2019) 28:717–32. doi: 10.1111/jocn.14696, 30357997

[8] McCaughan D Sheard L Cullum N Dumville J Chetter I. Patients' perceptions and experiences of living with a surgical wound healing by secondary intention: a qualitative study. Int J Nurs Stud. (2018) 77:29–38. doi: 10.1016/j.ijnurstu.2017.09.015, 29031127 PMC5744862

[9] Barbosa DR Arato CVB Bastos RA Reche NSG Oliveira MC Turato ER . Qualitative study on experiences reported by people with complex wounds in primary health care. Rev Bras Enferm. (2026) 79:e20240628. doi: 10.1590/0034-7167-2024-0628, 41739673 PMC12933937

[10] Tong A Sainsbury P Craig J. Consolidated criteria for reporting qualitative research (COREQ): a 32-item checklist for interviews and focus groups. Int J Qual Health Care. (2007) 19:349–57. doi: 10.1093/intqhc/mzm042, 17872937

[11] Francis JJ Johnston M Robertson C Glidewell L Entwistle V Eccles MP . What is an adequate sample size? Operationalising data saturation for theory-based interview studies. Psychol Health. (2010) 25:1229–45. doi: 10.1080/08870440903194015, 20204937

[12] Morrow R Rodriguez A King N. Colaizzi’s descriptive phenomenological method. Psychologist. (2015) 28:643–4. doi: 10.1016/j.jcin.2015.03.004

[13] Reinken DN Reed SM. Mishel's uncertainty in illness theory: informing nursing diagnoses and care planning. Int J Nurs Knowl. (2023) 34:316–24. doi: 10.1111/2047-3095.12406, 36366808

[14] Teklemariam BT Biyana CF Asfaw SA. Determinants of postoperative abdominal wound dehiscence among patients operated in a tertiary hospital. Ethiop J Health Sci. (2022) 32:739–46. doi: 10.4314/ejhs.v32i4.10, 35950069 PMC9341029

[15] Kunwor S Sapkota S. Post-thoracic surgery nursing care: a comprehensive review. Nepalese J Cancer. (2025) 9:97–103. doi: 10.3126/njc.v9i2.84912

[16] Hackert B Weger U Stuermer EK. Degrees of freedom-exploring unrecognised patient-empowerment in chronic wounds settings. J Wound Manag. (2024) 25:94–102.

[17] Seaton PCJ Cant RP Trip HT. Quality indicators for a community-based wound care Centre: an integrative review. Int Wound J. (2020) 17:587–600. doi: 10.1111/iwj.13308, 32030879 PMC7949261

[18] Vaismoradi M Rae J Turunen H Logan PA. Specialized nurses’ role in ensuring patient safety within the context of telehealth in home care: a scoping review. Digital Health. (2024) 10:20552076241287272. doi: 10.1177/20552076241287272, 39381815 PMC11459674

[19] Treger I Kosto A Vadas D Friedman A Lutsky L Kalichman L. Crafting the future of community-based medical rehabilitation: exploring optimal models for non-inpatient rehabilitation services through a narrative review. Int J Environ Res Public Health. (2024) 21:1332. doi: 10.3390/ijerph21101332, 39457305 PMC11508180

[20] Huang Y Hu J Xie T Jiang Z Ding W Mao B . Effects of home-based chronic wound care training for patients and caregivers: a systematic review. Int Wound J. (2023) 20:3802–20. doi: 10.1111/iwj.14219, 37277908 PMC10588341

[21] Monaro S Pinkova J Ko N Stromsmoe N Gullick J. Chronic wound care delivery in wound clinics, community nursing and residential aged care settings: a qualitative analysis using Levine’s conservation model. J Clin Nurs. (2021) 30:1295–311. doi: 10.1111/jocn.15674, 33506537

[22] Beeckman D Cooper M Greenstein E Idensohn P Klein RJ Kolbig N . The role community-based healthcare providers play in managing hard-to-heal wounds. Int Wound J. (2024) 21:e14402. doi: 10.1111/iwj.14402, 37715348 PMC10788587

