# Supplementary material for: Self-management perceptions and needs in patients with thoracic surgical wound dehiscence: a descriptive phenomenological qualitative study
Source: Front Public Health. 2026 Sep 30;14:1948457. doi: 10.3389/fpubh.2026.1948457 (PMC13661568; doi:10.3389/fpubh.2026.1948457)
Supplement: Supplementary file 1 [file Table_1.DOCX]

**S1 General Information**

| **Number** | **Gender** | **Age** | **Educational Level** | **Residence** | **Employment status** | **Primary disease** | **Patient source** | **Splitting time (day)** | **Occurrence of infection** | **Splitting part** | **Readmission** |
| --- | --- | --- | --- | --- | --- | --- | --- | --- | --- | --- | --- |
| 1 | Male | 68 | Middle school | Urban | Retired | Lung cancer | Outpatient | 14 | No | Right thoracotomy incision | No |
| 2 | Female | 55 | Middle school | Urban | Employed | Lung cancer | Inpatient | 5 | No | Right VATS mini-incision | Yes |
| 3 | Male | 72 | Primary school | Rural | Farmer | Esophageal cancer | Outpatient | 34 | Yes | Right thoracotomy incision | No |
| 4 | Male | 62 | Undergraduate | Urban | Retired | Mediastinal tumor | Outpatient | 21 | No | Median sternotomy incision | No |
| 5 | Female | 48 | Undergraduate | Urban | Employed | Lung cancer | Inpatient | 8 | No | Left VATS port site | No |
| 6 | Male | 59 | Middle school | Town | Self-employed | Lung cancer | Outpatient | 28 | Yes | Left thoracotomy incision | No |
| 7 | Female | 75 | Primary school | Rural | Farmer | Esophageal cancer | Outpatient | 37 | No | Right thoracotomy incision | No |
| 8 | Male | 65 | Middle school | Rural | Farmer | Pulmonary bullae | Inpatient | 6 | No | Right VATS mini-incision | Yes |
| 9 | Male | 53 | Undergraduate | Urban | Employed | Mediastinal tumor | Inpatient | 13 | Yes | Median sternotomy incision | No |
| 10 | Female | 60 | Middle school | Town | Retired | Lung cancer | Outpatient | 18 | No | Left VATS mini-incision | No |
| 11 | Male | 78 | Primary school | Town | Retired | Esophageal cancer | Outpatient | 52 | Yes | Right thoracotomy incision | No |
| 12 | Male | 67 | Middle school | Urban | Retired | Lung cancer | Outpatient | 17 | No | Right thoracotomy incision | No |
| 13 | Female | 52 | Middle school | Town | Self-employed | Mediastinal tumor | Outpatient | 14 | No | Median sternotomy incision | No |
| 14 | Male | 45 | Middle school | Urban | Employed | Severe chest trauma | Outpatient | 19 | No | Left thoracotomy incision | No |
| 15 | Female | 69 | Primary school | Rural | Farmer | Lung cancer | Outpatient | 32 | Yes | Right VATS mini-incision | No |
| 16 | Male | 61 | Middle school | Town | Retired | Esophageal cancer | Outpatient | 22 | Yes | Right thoracotomy incision | No |
| 17 | Female | 71 | Primary school | Rural | Retired | Lung cancer | Outpatient | 16 | No | Right VATS port site | No |
| 18 | Male | 56 | Undergraduate | Urban | Employed | Lung cancer | Inpatient | 6 | No | Left VATS mini-incision | No |
| 19 | Male | 74 | Middle school | Town | Retired | Esophageal cancer | Outpatient | 43 | Yes | Right thoracotomy incision | No |
| 20 | Female | 64 | Primary school | Rural | Farmer | Mediastinal tumor | Outpatient | 24 | Yes | Median sternotomy incision | No |
| 21 | Male | 58 | Middle school | Urban | Employed | Lung cancer | Outpatient | 19 | No | Right thoracotomy incision | No |

Note: VATS = Video-Assisted Thoracoscopic Surgery.
